# Leptospirosis in India: insights on circulating serovars, research lacunae and proposed strategies to control through one health approach

**DOI:** 10.1186/s42522-024-00098-5

**Published:** 2024-06-07

**Authors:** Baby Karpagam Krishnan, Ganesh Balasubramanian, Pesingi Pavan Kumar

**Affiliations:** 1 Department of Health Research (D.H.R.), ICMR-National Institute of Epidemiology (ICMR-NIE), Indian Council of Medical Research, Ministry of Health & Family Welfare, Government of India, R-127, 2Nd Main Road, T.N.H.B. Layout, Ayapakkam, Chennai, Tamil Nadu 600 077 India; 2 Department of Veterinary Public Health and Epidemiology, Faculty of Veterinary and Animal Sciences, Rajiv Gandhi South Campus, Banaras Hindu University, Mirzapur, UP 231001 India

**Keywords:** Leptospirosis, Humans, Animals, Environment, India, One Health approach, Epidemiology

## Abstract

Leptospirosis is one of the most neglected zoonotic infections of public health concern worldwide and a remerging infection in tropical countries such as India. The infection least explored disease and the epidemiological and other critical data are scarce for the disease rate reported and to control the infection. Leptospirosis as sapronosis is as underrated as the infection itself, and this article aims to explore the significance of this aspect of the disease. The research review aimed at the epidemiological understanding of the infection to control the negative impact of the disease. A mixed review and analysis were carried out to understand the knowledge published on the critical and understudied areas like epidemiology, transmission, diagnosis, treatment, and control of infection. A systematic analysis was carried out to extract information about the reported circulating strains, and research lacunae in India with the published data available in PubMed. The article elaborately discusses crucial inference areas of infection transmission and addresses lacunae in critically unacclaimed areas of infection to control the spread of infection using one health approach (OHA), and strategies to control leptospiral infection are proposed. The article also reviewed how and why Leptospirosis can be best studied and controlled by “One health approach” in India.

**Graphical Abstract:**

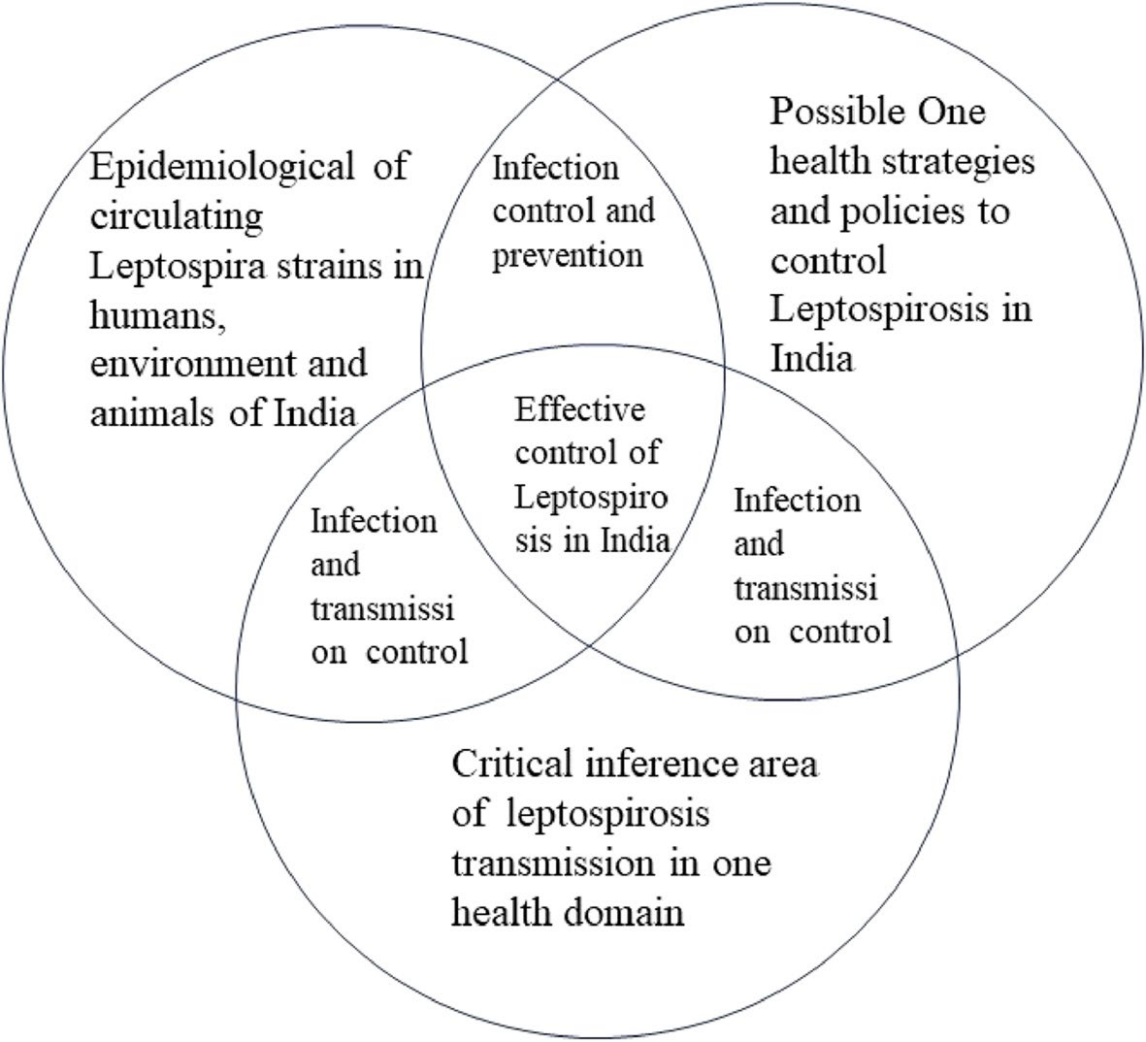

## Highlights

Leptospiral strains reported from one health domain source, human, animal, and environmental samples in India are listed to display the epidemiology of infection in India. This article discusses the lacunae in Leptospirosis research in crucial domains and one of the core areas of transmission and how it is the major target for the control of infection in animals and humans. The study has a list of recommendations for the establishment of a One Health working group and possible control strategies for transmission.

## Introduction

Approximately 60% of human infections are zoonotic, and 75% of these zoonotic infections are emerging and remerging [1, 2]. Leptospirosis is one of 17 neglected tropical infections listed by the World Health Organization [2]. It is an endemic infection in tropical countries and in some temperate regions and is prevalent globally [3]. The disease cycle of the infection is complex and dynamic, comprising humans, animals, and contaminated soil and water bodies. Hence, this zoonoses requires a holistic approach to the control and prevention of infection [4]. The infection is not adequately understood in an endemic country like India, to control and prevent the infection. Hence, intervention or policy-making for controlling this disease with an intricate transmission cycle and epidemiology must involve distinctive and thriving approaches to control and prevent infection [5].

“One Health” (OH) is a holistic approach that has been in practice dating back to the 1800s and lately is gaining popularity among researchers due to the effectiveness of the concept of controlling diseases, especially sapronosis. The concept addresses and identifies the domains that are involved in the transmissions and domains/factors that complement the persistence of pathogens. This approach encompasses human, animal, and environmental health and facilitates better control by providing balanced significance to dominating areas(s) that contribute to the spread and burden of infection threats to public health [6–8]. The current study aimed to identify the epidemiology of the infection in India with the reported strains, the research gap, and the effective areas of infection to control infection involving multiple mediums involved in infection.

Leptospirosis is prevalent globally, and it has been estimated that over 1 million infections occur in humans, with a mortality rate of 60,000 deaths annually [9]. The disease has slowly created a sense of public health emergency among the scientific community towards control due to the sudden surge and reemergence of infection [10]. Leptospira is one of the major pathogens that cause A.U.F.I. (acute undifferentiated febrile illness) in humans, infects productive animals, and is majorly present in the environment soil, and waterbodies. The infection contributes to a 2.90 million D.A.L.Y score per year, which is relatively more than other tropical infections, such as malaria, tuberculosis, filariasis, rabies dengue, etc. [11, 12]. Leptospira is a thin, long, slender hair-like, gram-variable spirochete [13]. There are 30 serotypes, and more than 350 serovars of leptospires have been reported to date categorized into pathogenic, intermediate, and saprophytic groups based on the pathogenicity [14].

In humans, it is primarily transmitted from infected, and carrier animals, they also shed bacteria in the environmental soil and water bodies from where humans contract the infection. This indirect mode from “environmental reservoir” is reported more than direct infection from animal sources. The entry of pathogens is reported to be through cuts or abrasions, inhalation, ingestion, and rarely through vertical and interhuman transmission [14–16]. The infection ranges from a mild flu-like illness to life-threatening multiorgan failure and death. Common clinical manifestations, such as fever, arthralgia, myalgia, typical frontal headache, pain and tenderness confined to calf muscles, conjunctival suffusion, lymphadenopathy, and occasionally skin rashes, are seen in 90—95% of cases, and severe infections are reported in the remaining 5–10% of cases infecting the hepatorenal system, termed “icteric” infection. Additionally, the delay in antibiotic treatment could lead to infection in the respiratory system. Another typical and unnoticed sign of this infection is conjunctival suffusion or uveitis in patients. 2) The icteric phase involves the liver and causes hepatic infection, resulting in jaundice [17–19]. Recently, the disease was found to manifest varied atypical and nonspecific clinical signs due to changes in the molecular material and geographical origin of strains [20]. The pathogen forms a biofilm in the epithelial cells of renal tubules and survives there for many months, shedding in urine, and acts as the major transmission host [21, 22].

In animals, the infection continues to be of utmost economic importance, directly influencing the country's economy through its impact on productive livestock. There is a wide range of domestic and wild animal hosts for transmission and maintenance of the pathogen. Major animals found to be affected are bovines, equines, porcines, rodents, etc. Infecting bovines, of all animals, has the greatest impact on the country's economy, as they are interrelated and impact agricultural and livestock activities [23]. In cattle and goats, reports of infection and its impacts indicated stillbirths, weak siblings, premature birth, abortion, sterility, mastitis, decreased milk productivity, etc. [24, 25]. The infection like in humans ranges from mild to life-threatening in ruminants and manifests through fever, anemia, and jaundice. Despite sharing equal risk with other animals, the epidemiology of leptospirosis in swine is not well established [26–28].

Leptospires affect mammalian hosts to maintain their transmission cycle. Rats are permanent and lifetime carriers of pathogens in the renal tubules of the kidney and can shed approximately 10^7^ to 10^8^ leptospires into the environment per ml of urine and in their body fluids [29, 30]. Other animals were found to secrete 5.1 × 10^8^ to 1.3 × 10^9^ cells per day and 5.1 × 10^9^ cells per day rats, as per a meta-analysis conducted by Barragan et al. [29]. Rodents are believed to be the carrier for diverse serogroups of Leptospira interrogans and Leptospira borgpetersenii serovars were also isolated from rodents in India [25].

Canines are notable transmitters of infection, chiefly, posing as asymptomatic infections where symptomatic infection presents with fever, lethargy, shivering, muscle tenderness, change in urination amount, dehydration, and loss of appetite. Depending on the mode of transmission, vomiting, diarrhea, indigestion, and dyspnea are caused by inhalation. The infection is mild, and the animals recover spontaneously with transient illness. Sparsely, kidney and liver infections are reported with jaundice and other life-threatening infections. Felines, however historically known to be resistant to Leptospirosis, were now reported with the infection due to the evolution of pathogens [31–33]. Wild faunal species such as deer, elephants, mongoose, and fish are also carriers of leptospires. The animal reservoir of leptospires in an area can be associated with the serovars in circulation. In other words, the serogroup of leptospires is confined to host animals.

Environmental waterbodies and soil play crucial roles in the existence, survival, and transmission of pathogens and are understudied, underestimated, and underrated [34–36]. Pathogenic Leptospires washed away from the soil with fertilizer during natural disasters like heavy rain and flooding, can increase the alkalinity of water bodies, and promote the growth and spread of disease-causing pathogens, posing a dual threat to ecosystems. Agricultural lands with stagnant water and wet soil reinforce the pathogens to thrive more and act as a source for the transmission of infection in the major occupational risk group, agricultural field workers [37]. Recent studies have indicated that leptospires can aggregate and aggregate with other bacteria, such as *Azospirillum brasilense, Sphingomonas, and Micrococcus,* to survive and exist against environmental stressors. Survival and multiplication of leptospires are complemented by the pH, soil moisture, minerals, and salt concentration of the environment [38, 39]. Leptospira has been found in various water sources, including freshwater bodies, agricultural areas, sewage systems, ornamental water features, and even the ocean. While pathogenic and intermediate strains need a host to spread the infection, the saprophytic biflexa group can cause infection without relying on a transmission host, as it feeds on organic matter [34–36, 40, 41].

### Methodology

Leptospirosis is endemic to India due to a tropical climate that complements the transmission of infection. Scientists believe that the first disease outbreak in the 1920s among convicts of Andaman with a pulmonary hemorrhage outbreak three years after the successful culturing of organisms [41]. To understand the circulating serogroups and knowledge gap we planned to carry out mixed methods to acquire information on the reported circulating serovars of the pathogens like systematic review and literature review. We collected 566 articles available in PubMed with the keywords “Leptospirosis” and “India” published until February 2023 systematically. The articles that published research data about circulating serovars through serological and molecular techniques were included. Data extraction was done for information on authors, year of publication, study region, the technique used for the identification, and source of sample without geographical restrictions and characteristics. With this systematic analysis and literature review of the research gap analysis, data on the circulating serogroups in humans, animals, and the environment were tabulated and discussed in the respective sections. The proposed intervention for the control of infection concerning the research gap also was given concerning the OH concept.

## Results and discussion

The literature review indicates that Leptospirosis was first suspected among convicts in the Andaman and Nicobar Islands in the early 1900s. However, critical aspects such as disease patterns, transmission mechanisms, and pathogenesis still lack comprehensive understanding. The spatial–temporal characteristics of the Indian subcontinent contribute to its status as a leptospirosis hotspot [36, 42]. Most of the serogroups of L. interrogans found in animals are known to cause infections in humans, but the reverse scenario is not reported. Also, other pathogenic species of Leptospira than interrogans in the analysis are not found except for the rodent population. Human infection from animal sources was reported, but the transmission pattern and the dynamics among animals, humans, and the environment are unclear. Understanding the transmission of infection and the role of different domains in zoonotic infections is crucial to determining the disease rate. Our review found that few studies studied two domains of the disease cycle and no study encompasses all three major domains of infection, hence the transmission pattern of the infection cannot be defined in India among animals, humans, and the environment [43, 44].

Spatially, Southern states of India, particularly Tamil Nadu, Karnataka, Kerala, and Puducherry, are endemic for leptospirosis, with a high number of reported cases. The Andaman and Nicobar Islands are notable hotspots for both infections and reporting on leptospirosis. Conversely, Maharashtra, Gujarat, Kolkata, and Orissa are also endemic, but with less reported information on leptospirosis [45–47]. Research articles on epidemiology show that over 50% of studies originate from Tamil Nadu, Kerala, Karnataka, Andhra Pradesh, Orissa, and the Andaman and Nicobar Islands. While the prevalent serogroups historically included were still found to infect the hosts, recent years have seen a shift in serogroups affecting both humans and animals [28, 48, 49].

Generally, humans are the major studied research domain in zoonotic diseases; in contrast, this research found that animal leptospirosis is a well-studied domain over humans in India. On the other hand, infection status in the human and animal populations needs to be studied well for the effective control of infection. Apart from domestic animals, the role of wild animals, especially at the human and wildlife ecological interface, crucial role in maintaining leptospiral infection has yet to be explored [50]. Environmental studies on leptospirosis are the least explored domain of zoonotic pathogens in India. [51–53]. Detecting and surveilling leptospirosis is complex and requires expertise, making the infection often overlooked due to insufficient awareness and negligence among healthcare workers, risk groups, veterinarians, public health scientists, and epidemiologists [23, 45].

To address the shortcomings of details about the epidemiology of leptospirosis in India, we also systematically analyzed the literature to identify the serovars circulating in India with the reported research. The Leptospira serovars reported in India from 1992 to date in animals and humans in different regions are shown in Table 1 [24, 32, 45, 54–62].

**Table 1:** Prevalent serogroups of leptospira in india reported by researchers around the country

SL No	State/Region	Reported/published year	Reference	Research Population	Predominant Serogroups
1	Uttar Pradesh	2022	[63]	Human	Lai, Hebdomadis, Bangkinang and Pomona
2	Agra	2021	[64]	Animals (Sloth bears)	Pyrogenes, Icterohaemorrhagiae, Javanica, Grippotyphosa, Canicola, and Tarassovi
3	North-east Province and Tamil Nadu	2021	[65]	Animals	Ballum, Grippotyphosa
4	Andaman & Nicobar Island	2018	[54]	Humans	Icterohaemorrhagiae, and Grippotyphosa
5	Tamil Nadu, Chennai	2018	[55]	Humans	Australis, and Autumnalis
6	India	2018	[24]	Bovine	Hardjo, Pyrogenes, Canicola and Javanica, Hebdomadis, Shermani, Panama, Djasiman, Tarassovi, Grippotyphosa, Pomona, Icterohaemorrhagiae, Copenhageni, Australis, Kaup, Hurstbridge, Bankinang, and Bataviae
7	India	2017	[66]	Rodents, Humans, and Bovine	Australis, Icterohaemorrhagiae, Autumnalis, Javanica, Icterohaemorrhagiae, Pomona and L. borgpetersenii in rats
8	Tamil Nadu, Chennai	2016	[67]	Humans	Icterohaemorrhagiae
9	South India	2016	[68]	Rodent and human	Autumnalis
10	Tamil Nadu	2016	[69]	Rodents, Humans and Bovine	Leptospira borgpetersenii serovar Javanica, Autumnalis
11	India	2015	[70]	Humans	Australis
12	Maharashtra	2014	[46]	Animals	Pyrogenes, and Icterohemorrhagiae
13	Tamil Nadu, Chennai	2011	[57]	Humans	Australis, Autumnalis, Canicola, Icterohaemorrhagiae, Patoc, and, Grippottyposa
14	Puducherry	2010	[58]	Humans	Icterohaemorrhagiae, Pomona, and Pyrogenes
12	Andaman & Nicobar Island	2010	[71]	Rodents	Autumnalis, Javanica, Icterohaemorrhagiae, Pomona, and Javanica. Leptospira borgpetersenii
13	Maharashtra	2009	[72]	Humans	Icterohaemorrhagiae, Bataviae, Tarassovi, and Pomona
14	Andhra Pradesh	2007	[73]	Humans	Icterohaemorrhagiae, Australis, Autumnalis, and Javanica
15	South India	2007	[31]	Animals	Autumnalis, Akiyami, and L. borgpetersenii Javanica Veldrat Batavia 46
16	Andaman & Nicobar Island	2006	[74]	Humans	Grippotyphosa and Australis
17	Odisha	2004	[75]	Humans	Canicola, Pomona and Hebdomadis
18	Andaman & Nicobar Island	2004	[76]	Humans	Grippotyphosa, Australis, Icterohaemorrhagiae, Hebdomadis, Canicola, and Sejroe
19	India	2003	[77]	Humans and Animals	Icterohaemorrhagiae, Hardjo, Patoc, Australis, Canicola, Grippotyphosa, Pyrogens, Pomona, Tarasovi and Ballum
20	North India	2003	[78]	Humans	Autumnalis, Icterohaemorrhagiae, Canicola, and Javanica
21	Tamil Nadu, Chennai	2003	[79]	Humans	Icterohaemorrhagiae
22	Andaman & Nicobar Island	2003	[80]	Humans	Valbuzzi
23	Andaman & Nicobar Island	2003	[81]	Rodents and Bovine	Grippotyphosa
24	Maharashtra	2002	[82]	Humans	Copenhageni
25	Tamil Nadu, Chennai	2000	[32]	Rodents and human	Icterohaemorrhagiae, and, Autumnalis
26	Kerala	1997	[83]	Humans	Autumnalis, Australis, Icterohaemorrhagiae, and, Australis bharathy
27	Tamil Nadu, Madras	1995	[84]	Humans	Autumnalis
28	Andaman & Nicobar Island	1995	[85]	Humans	Grippotyphosa, Canicola and JEZ Bratislava
29	Tamil Nadu, Madras	1993	[86]	Humans	Icterohaemorrhagiae, Panama, and Canicola
30	Tamil Nadu, Chennai	1992	[62]	Animal and human	Autumnalis

With the systematic analysis, we also found that the serovars specifically affected hosts from the data of included studies and presented in Table 1. With the mixed review, we found that Critical data on the infection like epidemiology, and transmission patterns and dynamics, the pathogenicity of the infection in humans and animals, the ability of the pathogen against environmental stressors are not reported. It is evident from the data Table 2 that L. interrogans is the most commonly reported species found to infect humans and in animals, serovars affecting cattle are reported more may be attributed to their economic impact on the country. It is also observed that the rodents carry diverse species including L. borgepetersenii, a human infection that is not reported in India [71]. A considerable number of investigations utilized MAT for the diagnosis by detection of antibodies against Leptospira infection using reference Leptospira serovar panel, molecular detection report is sparse. Though MAT is a sophisticated and cumbersome technique it is the basic and efficient tool in the diagnosis of leptospirosis [87]. Molecular typing techniques used were FAFLP and MLST with the STs of Leptospira strains reported in India are ST145 and ST27 from Tamil Nadu, which again proves that the data on molecular epidemiology is limited [69, 76].

**Table 2:** Leptospira serogroups reported and their hosts in India

No	Reservoir/carrier/ infected Hosts	Serovars
1	Cattle	Icterohaemorrhagiae, Harjo, Pyrogens, Canicola Javanica, Hebdomadis, Shermani, Panama, Djasiman, Tarassovi, Grippotyphosa, Pomona, Icterohaemorrhagiae, Copenhageni, Australis, Kaup, Hurstbridge, Bankinang, Patoc and Batavia of L. interrogans
2	Rodents-rats and mice	Autumnalis, Javanica, Icterohaemorrhagiae, Pomona, Grippotyphosa, Ballum, Australis serogroups of Leptospira interrogans and Javanica, Javanica, and Veldrat Batavia 46 of Leptospira borgpetersenii
3	Pigs	Pomona and Tarrasovi of Leptospira interrogans
4	Sheep	Pomona and Hardjo of Leptospira interrogans
5	Dogs	Canicola of Leptospira interrogans
7	Sloth bears	Pyrogenes, Icterohaemorrhagiae, Javanica, Grippotyphosa, Canicola, and Tarassovi of Leptospira interrogans
6	Humans	Lai, Hebdomadis, Bangkinang, Pomona, Icterohaemorrhagiae, Australis, Autumnalis, Canicola, Valbuzzi, Sejroe, Copenhageni, Australis bharathy, JEZ Bratislava, Panama, and Grippotyphosa of Leptospira interrogans

Table 1 projects the epidemiology by presenting the serovars circulating in India over time and there is not much change found in their trends over time through systematic search and data analysis. Recently, *Leptospira borgepetersenii,* a non-native serogroup in India, has been isolated from rats [69]. Nevertheless, its zoonotic potential has not been explored to advance the knowledge of the transmission of the infection [88]. Regarding collaboration, there is limited recognition of interdisciplinary, multi-center, international, and public–private collaborations in Leptospirosis. Limitations in the number and heterogeneity of research data exacerbate the research gaps. Hence we propose,

### One health coordination committee to understand transmission and improve diagnosis and surveillance of infection

Establishing an influential one health committee (O.H.C.) with profound knowledge and interest is the critical prospect of controlling infectious disease. Ideally, it should comprise veterinarians, environmental biologists, healthcare workers, physicians, army officers, social workers, and data scientists. An ideal OH concept should pass the following criteria: 1) As far as leptospires are concerned, the process should begin with the development methodology and conceptual framework that attributes detection, surveillance, and regular monitoring of the infection rate in humans and animals within an environment to get the basic knowledge about the epidemiology of infection in India [61, 89]. 2) The data collected from the previous process should be transferred to all the members of the committee to share ideas, concept modifications, and changes in methodology. 3) Acquisition of current knowledge about the interference of three domains and the epidemiological data obtained policies should be designed in a manner that welcomes research knowledge from every member of the committee. This team should validate the M.A.T. panel used in each region. The LipL32 is employed to detect pathogenic leptospires by serological and molecular detection must be revised with the more sensitive SecY gene, which should be further proceeded by updating the team alongside imposing the necessary protocol to be followed, wherever needed [90]. Appropriate intervention strategies include a) using appropriate PPE to risk groups that hinder the propagation of infection from the water-host interface, followed by preventative strategies towards control, sterilization, and vaccination of reservoirs against opportunistic carriers such as rodents and domestic animals such as cattle, dogs, goats, pigs, and wild animals [68, 74]. B) Vaccine design devised following the vast epidemiological knowledge acquired about infections among humans and animals. C) Approach towards altering the diagnosis criteria with an updated clinical presentation and diagnostic techniques to limit against the imposed differential diagnosis [91]. D) Minimize or clear misconceptions and propagate misleading information about the infection among and other than committee members. 4) At this point, the committee must ensure that devising policies, processes, and protocols are effective. The data should be circulated among them, considering every member to be significant and to be treated unanimously, irrespective of their domain. 5) Creating awareness about the infection by training social workers to create awareness among the target population [92]. 6) The committee should monitor the process continuously and update the process whenever required and wherever necessary until the goal of the committee is reached. 7) It should also keep an eye on the nonendemic area for any leptospirosis outbreak and control using doxycycline as a chemoprophylactic drug at the earliest to avoid mortality. Since little is known about the infection, the development of information technology and data science could be regarded as the foremost significant steps for O.H. processes, as it can be replaced by the following process for generating the current data acquired in real-time data [93].

### Forecasting disease transmission and epidemiology

Due to the inadequacy, data acquisition on the significant areas of the infection throughout the country especially in Northern India where the disease is least suspected due to the spatiotemporal characteristics. A vast pool of knowledge about the disease aspect of leptospirosis is beyond the bounds of possibility throughout India. Hence, forecasting the transmission rate and pattern will aid in defining whether there exists a similar condition/scenario or change in transmission reported in the Andaman and Nicobar Islands. This is feasible by employing computational biology tools such as ADEPTUS with the data acquired on the circulating serovars reported from different parts of India. [94–96]. Most importantly, this will leverage the achieved data on epidemiology and environmental reservoirs of leptospirosis, which needs crucial understanding. This will present scientists with knowledge about possible circulating serovars regionally and allow epidemiologists to make inferences and apply appropriate intervention strategies.

### Functional attributes of public/private organizations involved in disease control

Numerous control programs targeting leptospirosis have been conducted and documented. Expert researchers with an extensive understanding of the infection have advocated for various strategies, including surveillance enhancements and policy revisions, to curb its spread. In 2000, a collaborative effort was initiated, linking national centers across India, to establish a multi-task force aimed at estimating the disease burden associated with leptospirosis in the country. [97, 98]. Notably, an Informal Expert consultation on Surveillance, Diagnosis, and Risk Reduction of Leptospirosis held at ICMR-National Institute of Epidemiology conducted by WHO recommended a multisectoral and multidisciplinary approach to control infection back then in 2009. Following disease outbreaks, several endemic states like Kerala and Orissa established surveillance systems to monitor and respond to the disease but there is no Multi-centric, multidisciplinary, and National program throughout the country reported for surveillance and control [47, 99].

But in India, the presence of various boards overseeing different aspects of the One Health concept-encompassing humans, animals, and the environment, poses a challenge in coordinating committees aimed at controlling zoonotic diseases. Occasionally, these committees overlook the necessity of amending stringent national One Health policies for effective disease control [75]. There is a pressing need for an overarching organization to facilitate collaboration across these domains at a national level. Such an initiative is essential to combat the re-emergence, emergence, and persistence of infectious diseases. It is imperative to establish robust infrastructure, governance structures, and policies, allocate human resources, and provide effective leadership in India to effectively tackle the re-emergence of infectious diseases. [47, 100].

### Risk assessment and deduction

Understanding the infection rate and risk in one health domain will significantly aid in the control and prevention of reemergence of infection. In leptospirosis, the key and major area of transmission is where contaminated water surfaces interfere with human and animal hosts and should be monitored to observe whether this transmission rate enhances the transmission rate, infection rate, infection by multiple strains, etc. In India risk people who have a greater risk population of contracting the disease, including pets and domestic animals, should also be monitored for the same by trained veterinarians, healthcare providers, and medical officers without negligence.

Detecting and intervening with pathogenic leptospires, as well as identifying diversity among strains, will yield insights into local transmission at the community level. This knowledge is crucial for understanding the domains that facilitate and support transmission, addressing significant gaps in disease treatment. These data about the environment, animals, and humans at risk will help reduce the risk of infection. This should also include identifying the contaminated area and continuous, systematic environmental surveillance, as it is comparatively the least studied domain of one health approach in India and globally [101]. Abduction and treatment of suspected and confirmed animals with leptospirosis. Sterilization and vaccinating suspected permanent carriers such as rodents can be considered for the same. Proper sanitation and good hygienic practice training for the urban risk group of people living in slums can also control the infection [102].

### Proposed actions required by the public/private organizations

Collaboration of multiple sectors of public, private, and government bodies to control infection must be encouraged by organizations. It should ensure that the control program and intervention strategies implemented are followed up continuously through periodic discussion, continual review, and regular surveillance, including geographical and epidemiological aspects. It should support finances to disseminate the research information to the desired population through appropriate and trained professionals. Scientists, researchers, and activists should invest their knowledge equally in animals and the environment as humans and conduct “one health” research by providing fund schemes [103]. Public awareness education, providing PPE to the risk group, vaccinating animals, and controlling rodents will directly help strengthen India's economic status [104].

## Conclusion

Leptospirosis is a special and fastidious pathogen that requires sophisticated laboratory requirements for diagnosis and research; hence, it is traditionally neglected, and research data on the bacteria are scarce to control the infection [92]. The increase in the incidence of infection, emergence, and the reemergence of the disease in India could be attributed to the delayed report on infection, followed by a lack of process, and the medium of transmission as well followed by the lack of transferability of knowledge concerning the infection and its nature [105]. The review explored the lacunae to control infection and epidemiology in India. Epidemiology is crucial data to accurately diagnose and treat the infection and for the effective control of infection. Systematic analysis of data from the articles to extract cumulative data on circulating strains of pathogenic leptospires for the first time complements the comprehensive understanding and efforts to mitigate infection. Leptospira interrogans are the circulating genomospecies reported primarily from humans and animals. However, there is evidence of *Leptospira borgepetersenii* from rats, and their transmission to the other two domains of the disease requires more attention and exploration for a deeper knowledge of the epidemiology of the infection in India [69, 71]. The holistic, One Health approach will add insightful information on the crucial, dynamic, and significant areas of infection, transmission, and epidemiology of leptospirosis.

The study also discussed the mechanism through which pathogens in environmental stressors persist in infectious diseases, specifically zoonoses. However, as a sapronosis, the infection cannot be controlled only through conventional and regular strategies. Water bodies and soil/environmental domains act as the “environmental reservoirs” and key inference areas that complement the survival, maintenance, and proliferation of pathogens and the transmission of infection [106]. Hence, disease control intervention in this area of one health domain transmission should be considered pivotal and targeted with greater attention by emphasizing one health concept discussed for effective control of the infection.

OH lab aid towards diagnosis and surveillance of infection, followed by providing simultaneous or timely information, could effectively restrain the spread of infection in nonendemic regions [107]. Furthermore, it minimizes the cost of diagnosis, treatment, and prevention of disease by utilizing the supportive data imparted by OH. The database aids in sharing known data about infection. It provides room for trained professionals to report the most relevant and supporting data about a disease that will be established as a suitable approach in medical centers available in the vicinity of hotspots. Additionally, the database accessibility will be available for the public's disposal, which might plausibly step up for the betterment of repercussions.

## Data Availability

Not applicable.

## Abbreviations

OH: One Health
MAT: Microscopic agglutination test
PPE: Personal protective equipment
AUFI: Acute undifferentiated febrile illness
OHC: One health committee
FAFLP: Fluorescent amplified fragment length polymorphism
MLST: Multi Locus Sequence Typing
STs: Sequence Types ADEPTUS
